# The Role of Ionizing Radiation for Diagnosis and Treatment against COVID-19: Evidence and Considerations

**DOI:** 10.3390/cells11030467

**Published:** 2022-01-29

**Authors:** Marina Chalkia, Nikolaos-Achilleas Arkoudis, Emmanouil Maragkoudakis, Stamatis Rallis, Ioanna Tremi, Alexandros G. Georgakilas, Vassilis Kouloulias, Efstathios Efstathopoulos, Kalliopi Platoni

**Affiliations:** 1 2nd Department of Radiology, Medical Physics Unit, School of Medicine, National and Kapodistrian University of Athens, 12462 Athens, Greece; stamrallis@outlook.com (S.R.); stathise@med.uoa.gr (E.E.); polaplatoni@gmail.com (K.P.); 2 2nd Department of Radiology, Diagnostic Radiology Unit, School of Medicine, National and Kapodistrian University of Athens, 12462 Athens, Greece; narkoudis@med.uoa.gr; 3 2nd Department of Radiology, Radiation Oncology Unit, School of Medicine, National and Kapodistrian University of Athens, 12462 Athens, Greece; emmanouil.maragkoudakis89@gmail.com (E.M.); vkouloul@med.uoa.gr (V.K.); 4 DNA Damage Laboratory, Physics Department, School of Applied Mathematical and Physical Sciences, National Technical University of Athens (NTUA), 15780 Athens, Greece; ioannatremi@mail.ntua.gr (I.T.); alexg@mail.ntua.gr (A.G.G.)

**Keywords:** ionizing radiation, COVID-19, chest CT, imaging, low dose, radiotherapy, low dose radiation therapy (LDRT), anti-inflammatory treatment

## Abstract

The Coronavirus disease 2019 (COVID-19) pandemic continues to spread worldwide with over 260 million people infected and more than 5 million deaths, numbers that are escalating on a daily basis. Frontline health workers and scientists diligently fight to alleviate life-threatening symptoms and control the spread of the disease. There is an urgent need for better triage of patients, especially in third world countries, in order to decrease the pressure induced on healthcare facilities. In the struggle to treat life-threatening COVID-19 pneumonia, scientists have debated the clinical use of ionizing radiation (IR). The historical literature dating back to the 1940s contains many reports of successful treatment of pneumonia with IR. In this work, we critically review the literature for the use of IR for both diagnostic and treatment purposes. We identify details including the computed tomography (CT) scanning considerations, the radiobiological basis of IR anti-inflammatory effects, the supportive evidence for low dose radiation therapy (LDRT), and the risks of radiation-induced cancer and cardiac disease associated with LDRT. In this paper, we address concerns regarding the effective management of COVID-19 patients and potential avenues that could provide empirical evidence for the fight against the disease.

## 1. Introduction

The rapid spread of Coronavirus disease 2019 (COVID-19) has led to a pandemic, which has affected seriously almost all countries worldwide. COVID-19 is a big concern because it has high transmission rate, and significant mortality rate, something that seems to be changing rapidly though with the new Omicron variant, but at the same time the treatment options are limited. The mortality rate of COVID-19 is usually compared to that of the seasonal flu, as both can present severe respiratory symptoms. According to WHO estimates, 290,000 to 650,000 people die of flu-related causes every year worldwide, while over 5.5 million deaths from COVID-19 have been reported [1,2]. Most patients with COVID-19 exhibit mild-to-moderate symptoms, but approximately 15% progress to a severe “pneumonic” state, and approximately 5% eventually develop acute respiratory distress syndrome (ARDS), which leads to pneumonia and even respiratory failure [3]. For initial diagnosis of COVID-19, reverse transcription-polymerase chain reaction (RT-PCR) testing has been recommended as the gold standard, providing high specificity and being easily implemented by throat swab sampling. However, the ongoing pressure on healthcare facilities worldwide has urged the need for implementation of alternative diagnostic tools, such as Computed Tomography (CT) scans. Several approaches to treat COVID-19 have been advocated. Symptomatic management and oxygen therapy are two main clinical management options [4]. For the symptomatic management, a variety of pharmacologic options are available that include antiviral drugs (e.g., remdesivir), anti-SARS-CoV-2 monoclonal antibodies (e.g., bamlanivimab/etesevimab, casirivimab/imdevimab), anti-inflammatory drugs (e.g., dexamethasone), immunomodulators agents (e.g., baricitinib, tocilizumab) [5]. In the struggle to manage the continuously incoming patients on healthcare systems, scientists have suggested Low Dose Radiation Therapy (LDRT) as a possible therapy for COVID-19 patients [6,7]. With a total dose to the whole thorax ranging between 35 and 150 cGy, LDRT could be effective in reducing the inflammatory response [8]. These suggestions were initially based on a review from Calabrese, describing outcomes from LDRT for viral or bacterial pneumonia conducted in the pre-antibiotic era [9]. However, the evidence supporting its effectiveness is limited and of low quality due to the limitations both in the human data and in the biological data [10]. Moreover, the risk of radiation-induced cancer (RIC) and circulatory disease for the patients being treated should be assessed. As the methodology of radiation treatment for COVID-19 is new, measurement of potential long-term radiation risk is not yet possible. However, the large amount of data that have accumulated over decades for the effects of ionizing radiation allow quantitative estimates of potential risks of radiation treatment to be calculated.

## 2. Materials and Methods

### 2.1. Literature Search

A comprehensive literature search was performed in PubMed, ScienceDirect, and ClinicalTrials.gov. Search terms included ionizing radiation, COVID-19, chest CT, imaging, low dose, radiotherapy, low dose radiation therapy (LDRT), anti-inflammatory treatment. Articles retrieved were published in peer-reviewed journals and in the English language. No date restriction was applied.

### 2.2. Ionizing Radiation for Diagnostic and Therapeutic Purposes

Ionizing radiation is a form of energy emitted by atoms that travel as electromagnetic waves (gamma or X-rays) or particles (neutrons, beta, or alpha). Humans can be exposed to ionizing radiation by natural sources such as water, soil, and flora containing naturally occurring radioactive materials, natural radiation from cosmic rays, and also by human-made sources, spanning from nuclear power generation to medical application of radiation for diagnosis or treatment [11]. Due to its versatile nature, ionizing radiation can be variously utilized for diagnostic procedures in healthcare. The methods of its implementation in that direction can be classified into two categories. X-rays, mammography, CT scans, and fluoroscopy comprise the external diagnostic applications of ionizing radiation, while nuclear medicine is used for internally assessing diagnostic findings.

The chest X-ray (CXR) is a well-established and effective technique employed for medical purposes in order to assess the airways, the pulmonary parenchyma and vessels, mediastinum, and its structures (i.e., heart), pleura, and chest wall [12]. The common and accepted practice involves obtaining posteroanterior (PA) and left lateral radiographic images with the patient in the upright position. However, portable chest radiography may be preferred and indicated in certain clinical situations and patient populations (e.g., critically ill, postoperative, trauma, and newborn patients) and should be performed in those cases. The indications for performing a CXR are innumerable, ranging from the assessment of signs and symptoms that may be associated with the respiratory, cardiovascular, Upper Gastrointestinal (GI), or thoracic musculoskeletal systems, to the monitoring of known thoracic processes, to monitoring patients following cardiac, thoracic or other surgical procedures, and even to comply with government regulations (i.e., surveillance for active TB) [12].

A CT scan is a form of radiological imaging examination also exploiting ionizing radiation since it is essentially an X-ray examination. However, when a CT scan is performed, a series of X-rays are rotated around a specific portion of the body, producing computer-generated cross-sectional images. The merit of these tomographic images over conventional X-rays is that they provide detailed information concerning a particular area in cross-section, avoiding superimposition of structures, hence providing a significant advantage over plain X-rays [13]. Amongst various others, clinical indications for high-resolution chest CT include the assessment of suspected lung disease when CXR findings are normal, the clarification of the pattern of disease on CXR (in order to limit the differential diagnosis), and the assessment of the activity of disease [14].

Ionizing radiation can also be used for treatment purposes. Radiation creates ions as it passes through atoms and can kill cells or change the cells’ DNA so that the cells stop growing. Radiation therapy has become a standard of care for treating many types of cancer, as it exploits radiobiological differences between cancer cells and normal tissue cells [15]. Radiation therapy is used as a curative treatment, as a palliative treatment, and/or as adjunctive therapy. Nuclear medicine is also used for therapeutic purposes where radiopharmaceuticals emitting adequate energy are administered to kill hyperfunctioning or malignant cells (e.g., hyperthyroidism, cancer of the thyroid, palliative treatment of bone metastases). Besides the treatment of malignant tumors, radiation therapy is an accepted treatment for benign diseases, such as acoustic neuroma, meningiomas, trigeminal neuralgia, gynaecomastia [16]. A novel application of ionizing radiation for therapeutic purposes seems to be for the treatment of patients with COVID-19 [17,18]. Studies are expected to prove the benefit of this radiation therapy.

### 2.3. Ionizing Radiation for Diagnostic Purposes of COVID-19

More specifically, ionizing radiation can be employed in medical applications for the assessment and monitoring of COVID-19, where it can play an important supplementary role to RT-PCR tests, which are the gold standard for detection of this disease [19,20]. Combining various diagnostic tests with the RT-PCR tests could be the most efficient way to maximize sensitivity and specificity for COVID-19 diagnosis [20]. Such diagnostic tests include imaging techniques exploiting ionizing radiation in order to obtain diagnostic images of COVID-19 pneumonia-affected lung parenchyma [21,22].

#### 2.3.1. Τhe Role of Chest X-ray (CXR)

In everyday clinical practice, CXRs are an irreplaceable tool for lung parenchyma assessment representing the fastest, most widely available, and cost-effective imaging technique. Nevertheless, CXRs can be insensitive in mild COVID-19 infection or early in the course of the disease [23], thus resulting in false-negative radiographs [24,25,26,27]. Therefore, CXRs should not constitute the first-line imaging technique and should rather be retained for the bedside follow-up of intensive care unit (ICU) patients, who—due to their condition—may not be transferred to the CT suite [28]. Ultimately, the decision of which imaging modality should be used is left to clinical judgment and may be subject to local resources, availability, and expertise [23].

#### 2.3.2. The Role of Chest CT

Due to its higher sensitivity when compared to CXRs, the COVID-19 imaging literature is primarily focused on chest CT. Importantly, studies have suggested the possibility of chest CT to detect COVID-19 pneumonia imaging findings prior to an RT-PCR positive test [29]. Additionally, in epidemic areas with high pre-test probability, chest CT has been found to have comparable sensitivity to RT-PCR assays in diagnosing COVID-19 disease [30]. Despite its high sensitivity for COVID-19 pneumonia diagnosis, the CT findings of COVID-19 patients are not specific since they can be similar to those caused by several other noninfectious and infectious entities, including influenza viruses, H1N1, SARS, MERS, and organizing pneumonia [31,32,33].

As a result, due to the non-specific appearances, several radiological societies do not currently endorse utilizing chest CT for primary diagnosis of COVID-19 disease, nor for screening purposes [34,35,36]. When there is RT-PCR availability and patients exhibit symptoms of mild disease, most organizations do not support diagnostic imaging. However, if the availability of RT-PCR tests is limited, chest imaging may be employed in cases with moderate-severe COVID-19 pneumonia-related clinical features [33]. Moreover, it is advised that CT imaging is retained for patients who are hospitalized and/or symptomatic with certain clinical indications, as well as for those patients with suspicion of COVID-19 disease complications (i.e., abscess, empyema) [34,35,36]. Nonetheless, in everyday clinical practice, chest CTs may often be performed in the management of COVID-19 suspected and/or confirmed cases and may be used as a complementary diagnostic tool. Moreover, initial chest CT may often be included in the workflow of some centers mainly to enable patient triage [37].

#### 2.3.3. Scanning Considerations

Due to the fact that COVID-19 pneumonia findings are mostly limited to the lung parenchyma with only rare involvement of the pleura and mediastinum, there is only little use of iodinated contrast administration. As a result, for the evaluation of the majority of patients with COVID-19 infection, a non-contrast chest CT with an inspiratory breath-hold will be sufficient while a post-contrast chest CT will be helpful when there is clinical worsening of cardiorespiratory status, suspicion of pulmonary embolism, or suspicion of complicated disease [33]. Whenever possible, CT must be performed with an inspiratory breath-hold, and images should extend from the lung apex to the lung base.

According to the International Commission of Radiologic Protection (ICRP), the as low as reasonably achievable (ALARA) principle should be followed in a daily practice of radiology even in a pandemic situation [21]. That highlights the necessity for integration of low dose CT (LDCT) or Ultra Low Dose CT (uldCT) protocols into healthcare facilities’ daily routine workflow against COVID-19 worldwide [38,39]. As of now, many literature studies, presented in Table A1, have been conducted by utilizing LDCT or uldCT protocols.

In April 2020, the International Atomic Energy Agency (IAEA) organized a webinar regarding CT practice and optimization of protocols for COVID-19. In this workshop, it was pointed out that most low-dose chest CT protocols can be achieved at less (or equal) to 100 kVp and low tube current. Additionally, a Dual Energy (or Dual kVp CT) with LDCT protocol and Sn-100 filter may also be implemented [40]. Low tube current (mAs) may be set to low-fixed, but this requires attention in the case of some overweight patients. Correct implementation of automatic exposure control (AEC) is recommended, as it can lead to up to 40% dose reduction [41]. Users can target a volume CT dose index (CTDIvol) of lower than 5 mGy, which is recommended for lung cancer screening. The scan mode may be helical or spiral with high pitch and a fast rotation speed to deal with potential patients’ motions during the examination. In all aforementioned literature studies, LDCT and uldCT protocols have been implemented by utilizing non-contrast chest CT, with lower tube current, ranging from 20 mAs (uldCT) to 45 mAs (LDCT). Additionally, pitch factors were set as high, with values from 1.2 to 1.5. Rotation times ranged from 0.35 s to 0.75 s. The most frequent kVp values were 120kV followed by 140, 100, and 80 kV.

Although many literature studies agree with IAEA statements on the implementation of low dose (or ultra low-dose) protocols, there seems to be confusion on their utilization. A worldwide study on variations in CT utilization, protocols, and radiation doses was conducted by Homayounieh (2020). This study included the participation of 64 health sites across the world [42]. It was reported that CT examinations are mostly used to monitor the severity of disease and less for diagnostic purposes. However, in other reports, it is pointed out that there are still many countries using CT as a diagnostic tool for patients suspected of COVID-19, possibly due to limited resources [21,43]. Homayounieh’s main finding was variations in the number of scan phases and CT exams per patient among healthcare facilities across the world. Additionally, they ascertained variations among effective doses recorded from COVID-19 patients, with differences of approximately twenty times between lower and higher values [42].

#### 2.3.4. CT Imaging Findings

**Figure 1:**
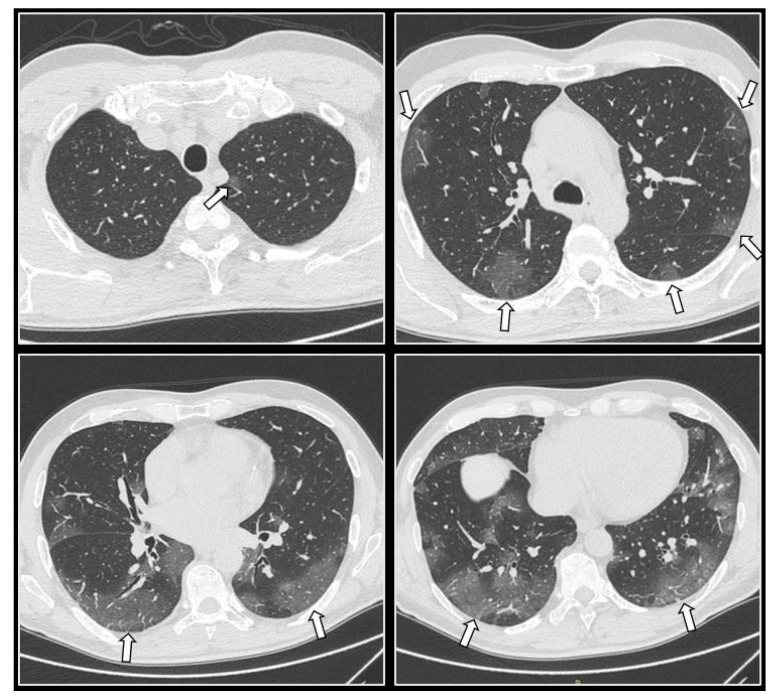
Axial images from a chest CT of a COVID-19 patient demonstrate multiple ground glass opacities (arrows) in both lungs and multiple lobes which are primarily distributed peripherally, posteriorly, and in the lower zones. Opacities occupy approximately 26–50% of the total lung parenchyma according to visual assessment [45].

COVID-19 pneumonia imaging findings on CT can range from typical to atypical with the most characteristic findings including ground glass opacities (GGOs). GGOs are located in both lungs and multiple lobes, and mainly distributed peripherally, posteriorly and in the lower zones (Figure 1), while common findings also include consolidations and crazy paving infiltrates (GGOs with concomitant intralobular or interlobular septal thickening) [31,32,44].

Furthermore, imaging findings on CT can also vary according to the course of the disease at the time of imaging. Notably, four stages on chest CT have been described according to the course of the disease: early on, at the initial stage (0–4 days), a chest CT may appear normal, or it may only demonstrate GGOs; then, at the progressive stage of the disease (5–8 days) an increase in GGOs may be displayed, while crazy paving infiltrates may appear as well. Subsequently, at the peak stage (9–13 days), consolidations can be demonstrated. Finally, at the absorption stage (>14 days) a gradual resolution of the findings may be initiated with “fibrous stripes” appearing and the abnormalities frequently—but not always—resolving at one month and beyond [44,46].

The available literature advocates that the quantification of COVID-19 disease extent, as perceived on CT, is associated with prognosis, and prediction of poor outcomes [28]. Numerous studies have been published on that matter with various quantification techniques being employed. Li et al. [47] assessed COVID-19 disease extent by quantifying the opacities in each of the five pulmonary lobes separately, whereas Yang et al. [48] divided the lungs into 20 bronchopulmonary segments and then evaluated each one of those segments individually for the presence of COVID-19 opacities. The authors proposed that visual quantification can parallel COVID-19 clinical classification [47] and that CT scoring is higher in severe than in mild cases [48].

On a similar note, other studies quantify the percentage of pulmonary parenchyma affected by the disease, by evaluating both lungs as a whole, while using a simple visual percentage scaling system with scales ranging from 0 to 100% (Figure 1) [45,49,50,51,52]. Specifically, in a study conducted by Guillo et al. [49], the great majority of patients demonstrating more than 25% occupancy of the total lung parenchyma by COVID-19 opacities were either intubated or deceased in the first 3 weeks after the initial CT. Similarly, Colombi et al. [50] demonstrated that patients with pneumonia-occupied parenchyma of >27% (well-aerated lung parenchyma of <73%) had a 5.4 times greater likelihood of dying or being admitted to the ICU. Furthermore, Ahlstrand et al. [52] found that the use of such a scoring system (0%, <10%, 10–25%, 25–50%, 50–75%, >75%) on admission CT could strongly predict in-hospital mortality and ICU admission. Grégory et al. [51] displayed that the extension of total pulmonary parenchyma affected by COVID-19 (<25%, 25–50%, 50–75%, >75%) could independently predict poor patient outcome and be correlated with the requirement of mechanical aeration and in-hospital mortality during the first 2 weeks. The above studies highlight the realization that obtaining and evaluating admission chest CTs based on the visually assessed burden of COVID-19 disease may predict ICU admission and in-hospital death, thus improving patient management and aiding in risk-stratification.

### 2.4. Ionizing Radiation for Treatment Purposes of COVID-19

#### 2.4.1. Low-Dose Radiation Therapy (LDRT) for COVID-19: Modulatory Effects

Historically, LDRT was used as an effective treatment for various inflammatory and infectious diseases, such as gangrene, sinusitis, arthritis, and pneumonia. X-rays were widely used against lower respiratory tract pathogens before World War 2, in the pre-antibiotic era in the USA. Calabrese reported cumulatively 717 cured cases of bacterial, interstitial, and atypical pneumonia, reported from studies of that era [9]. LDRT with photons at 50–100 rad in adults, and 35–40 rad in children, led to resolution of symptoms, improvement of biomarkers, and reduced mortality, with comparable mortality rates to sulphonamides and serum therapy. The turning point occurred with the discovery of penicillin in combination with increased cancer risk awareness from radiation, as a result of the nuclear bombing at the end of World War 2. These factors led to a loss of interest in LDRT for the treatment of pneumonia, further efforts were abandoned, and no new research papers have been published since 1946.

Until now, despite the numerous pharmacological studies, the most efficacious treatments for pneumonia are based on oxygen supplementation, either through a nasal cannula or mechanical ventilation. However, the use of such equipment, for example in intensive care units, is often limited. This difficult situation has raised the interest to reconsider the not very-well known historical treatment of patients with LDRT. The clinical rationale behind LDRT as a way to treat COVID-19 pneumonia derives from its anti-inflammatory action by inhibiting the so-called “cytokine storm” that leads to ARDS, respiratory failure, and eventually death. The two main suggested mechanisms by which radiation therapy mediates its clinical benefit are through nuclear factor erythroid 2-related transcription factor (Nrf2) activation which induces macrophages to an M2 anti-inflammatory phenotype and through direct anti-oxidative and anti-inflammatory effects [53].

Throughout history, the use of radiotherapy to treat pneumonia has been well documented and might provide valuable insights to current perspectives [9]. For instance, Calabrese’s review focuses on fifteen reports of various patient cases, which presented with severe pneumonia following bacterial or even viral infection [54]. These patients were treated with low doses of kilovoltage X-rays, which resulted in a reduced mortality rate and an overall improved clinical picture shortly 1–3 days after radiation. Moreover, no differences in the response rates were observed between bacterial or viral-induced pneumonia. However, these studies are originated from 1900 to ~1950 and, compared to recent clinical reports, they do not cover a large number of patients and many of them lack the appropriate control groups. In the following decades, no other reports were made regarding the use of low-dose radiotherapy to treat pneumonia, making it more difficult to introduce such a method into the clinic. However, one common feature of previous investigations is their effectiveness in doses ranging between 10 and 100 cGy shared with current schedules of radiation therapy for benign painful inflammatory degenerative disorders [55]. The biological mechanisms depicting the effectiveness of these low doses have been thoroughly researched and characterized in the recent years. Numerous in vitro and in vivo studies have evidenced the close relationship between low-dose ionizing radiation and inflammatory responses underlying mainly the contribution of leukocytes, macrophages, fibroblasts, and endothelial cells along with the secretion of cytokines/chemokines and growth factors [53,56]. The observed mechanisms seem to be common at the corresponding low doses, although their action is more effective in the range between 30 and 70 cGy. Even though there are no experimental or preclinical data to support the above-mentioned exhibits, a single dose of 50 cGy to the entire lung may be recommended based on radiobiological considerations. The main hypothesis for using LDRT to treat COVID-19 is that targeted radiation to the lungs will stop the uncontrollable inflammation that induces pneumonia which, in turn, dramatically worsens the course of some patients. Many studies suggest that inflammatory cascades in the infected lung tissues appear to be the main culprit for the severe illness and death were due to COVID-19 pneumonia [57,58,59].

An important factor that has to be taken into consideration before clinical application is the appropriate timing of irradiation during infection. Normally, at the early stages of a viral disease, the spread of the virus inside the host can be limited by type I interferons (IFNs-I), which inhibit viral replication. IFNs-I are secreted cytokines that activate signal transduction leading to the increased induction of interferon-stimulated genes acting as regulators of the innate and adaptive immune responses On the other hand, IFNs-I expression, when sustained, might alter the effectiveness of antiviral responses resulting in viral persistence [60]. Based on the above, the successful treatment of SARS-CoV-2 is directly dependent on the impending complexity and time frame of action of the immune system. In addition, it has been reported that, in severe cases of SARS, where there was extensive lung damage, high serum levels of pro-inflammatory cytokines and increased accumulation of innate immune cells in the lung were observed [61]. This was confirmed recently when, in acute cases of COVID-19, elevated levels of in-terleukin-6 (IL-6) in the serum were reported. An additional prominent risk factor for severe disease progression is d-dimer > 1µg/mL in the serum. Preferentially, endogenous protective immune responses at the incubation stage should be achieved by boosting the immune system with, e.g., INF-alpha or anti-sera [62]. The latter might even be effective in the severe phase of the disease [63]. Lung treatment with low dose radiation can significantly improve the patient’s levels of inflammation and slow down cornification, especially during the early to medium stages of SARS-CoV-2 infection. Moreover, low doses of radiation have been found to stimulate anti-viral immune defenses including natural killer cell activity and IFN production [64]. In contrast to early stages of the COVID-19 disease, advanced later stages are characterized by cytokine release syndrome (CRS, cytokine storm), and so LDRT might not be efficient anymore, since the systemic immune response progresses in a non-controlled manner. It is worth mentioning that, during the recovery phase of COVID-19 patients, activated CD38 and HLA-DR expressing CD4+ and CD8+ T cells are particularly increased alongside IgM and IgG SARS-CoV-2-binding antibodies [65]. Therefore, the timing of irradiation has to be decided with care in order to avoid attenuation of diseases resolving immune response, e.g., by stimulating IFNs-I.

Regarding now biological damage, previous experimental and simulation studies suggest that exposure for example of the thoracic area and infected lungs to an X-ray dose of 50 cGy is expected to induce a relatively low number of RNA damage and mutations in the virus and therefore a low selective pressure. More specifically, in a 30 kb virus single-stranded genome, one would predict for the specific dose: ~0.005 single-strand breaks-SSBs/virus (~1000 SSBs per a ~3 Gb genome) and 5–6 times more oxidative base lesions [66,67]. SARS-CoV-2 is a highly mutagenic RNA virus, even more so compared to the corresponding SARS RNA viruses of a human host [68]. Therefore, as has been already stated, the use of antiviral drug treatments against SARS-CoV-2 would probably result in a more intense selective pressure on the virus [69]. Under this pressure, in the near future, viruses that will survive will adapt and become even more resistant to any drug-based antiviral therapy and of high pathogenicity.

#### 2.4.2. LDRT Clinical Trials

Current studies have attempted to address issues of paramount significance related to the practical application of LDRT in the treatment of COVID-19 pneumonia, such as the degree of clinical benefit, potential risks, dose schedules, timing of radiotherapy delivery within the COVID-19 pneumonia cycle (early infection, pulmonary phase, hyper-inflammation phase), groups of patients that will benefit most, feasibility and cost-effectiveness. At the time of writing this review, all studies were phase I or II, with one of them transitioned to phase III (Table A2), while the ongoing ones are presented in Table A3.

Sanmamed et al. from Spain, conducted a phase I-II single-arm study (LOWRAD-Cov19) from April to June 2020, aiming to assess the radiological and clinical efficacy of LDRT against COVID-19 disease [17]. The primary endpoint was the radiological response and secondary endpoints were oxygen therapy dependence response, inflammatory markers response, hospital days, and acute toxicity. Patients included were older than 50 years old, at the pulmonary or hyperinflammation phase of the disease, and requiring oxygen therapy. The Clinical Target Volume (CTV) included both lungs and the Organs at Risk (OAR) contoured were heart and esophagus. The dose of LDRT was 100 cGy in a single fraction, with a 3D conformal technique, with Anterior–Posterior fields. A total of 9 patients with a median age of 66 received LDRT 52 days post-admission to hospital and 25 days after their last anti-COVID-19 treatment. Disease extension as per Chung scoring was significantly improved from the day of CT simulation to day 7 post-LDRT [70]. Oxygen requirements were significantly improved, as was Lactate Dehydrogenase (LDH). In terms of acute toxicity, there were two grade II lymphopenias 72 h post-RT and one patient with deteriorating grade III lymphopenia that progressed to grade IV a week following RT.

Ameri et al. from Iran designed a single-arm study that contained two different dose schemes aiming to assess the radiological and clinical efficacy of LDRT [18]. Their primary outcome was an improvement in oxygen saturation (S_P_O_2_) and secondary outcomes were hospital days, the number of intubations, 28-day mortality, and inflammatory biomarkers. Inclusion criteria were age (>60 years old), COVID-19 with moderate pulmonary involvement defined as Respiratory Rate (RR) more than 30 breaths per minute or S_P_O_2_ < 93% on room air. Dose schemes were for the first phase: 50 cGy in a single fraction plus another 50 cGy (100 cGy in total) at the physician’s discretion after a minimum of 3 days from the first fraction and for second phase: 100 cGy in a single fraction. Response was defined as S_P_O_2_ increase one day post-RT with subsequent improvement (Response rate—RR) and S_P_ O_2_ > 93% on room air on discharge (Clinical recovery—CR). A total of 10 patients were enrolled in total. Patients 1 to 5 received 50 cGy, patient 6 received 50 cGy another 50 cGy 6 days later due to deterioration (1st phase) and, finally, patients 7–10 received 100 cGy (2nd phase). RR was 63.6% with initial improvement in S_P_O_2_ in 90% of patients and CR was 55.5%. There were no significant differences in outcomes between the two-dose schemes.

Hess et al. from Emory University, USA, published the results of their phase I-II trial (RESCUE 1-19), from April until May 2020, and have now proceeded to the phase III component [71]. They aimed to evaluate the safety (phase I) and preliminary efficacy (phase II) of LDRT. Phase I included 5 cohort patients and a 7-day interim toxicity analysis (potential “storm” exacerbation), while phase II component included 10 patients who received LD-RT and were compared to 10 retroactively and blindly selected, matched controls. Inclusion criteria were patients requiring hospitalization for oxygen therapy, clinically deteriorating with consolidation on chest x-ray or computed tomography. Primary endpoint was Time to Clinical Recovery (TTCR) defined as off-oxygen therapy for more than 12 h. Secondary endpoints were hospital days, the number of intubations, duration of intubation, oxygen requirements, level of consciousness, radiological changes, and biomarkers. The dose was 150 cGy as a single fraction, with a 2D technique and a single beam of 15 MV energy delivered through an open anterior–posterior field. Mean age was 78 years for the LDRT group and 75 years for the control. Median TTCR was 3 versus 12 days favoring LDRT (*p*: 0.046) and delirium was improved with a Glasgow Coma Scale (GCS) 2.5 times higher in LDRT group (*p* < 0.01). All other endpoints, except for 28-day mortality, trended in favor of LDRT without reaching statistical significance, although 28-day overall survival was 90% for both groups. No serious adverse effects were reported (1 patient experienced grade 1 upper GI symptoms: nausea).

Papachristofilou et al. from Switzerland conducted a double-blind phase II trial (COVID-RT-01) comparing LDRT versus sham RT, enrolling critically ill patients, either intubated or on Non-Invasive Ventilation (NIV) [72]. They delivered 100 cGy in a single dose, with a 2D technique and beam energy of 10MV. The primary endpoint was Ventilation Free Days (VFD) at day 15 post-intervention. They hypothesized that LDRT would result in an increase of more than 10 VFD at day 15 compared to the Standard of Care as measured from in-house data: VFD at D15: 3.93 days. A total of 22 patients were enrolled with a median age of 75 years. The median VFD on day 15 was 0 for both groups. Overall survival on days 15 and 28 was similar for both groups. Secondary endpoints were oxygen requirements, biomarkers, ARDS severity, and no differences were noted between the two groups. More pronounced relative reductions in lymphocyte counts were noted in the LDRT group (*p* < 0.01). This was the first randomized study of critically ill patients and the authors concluded that there is no benefit in this particular group. Authors underlined also that the 2D technique shortened their stay in the radiotherapy department, minimizing the risk of unexpected events outside ICU, at a cost of a less accurate treatment planning compared to more advanced techniques (3-Dimension Conformal Radiotherapy- 3D-CRT, Intensity Modulated Radiotherapy—IMRT, Volumetric Modulated Arc Therapy—VMAT, Image Guided Radiotherapy—IGRT).

Finally, Arenas et al. reported the largest number of patients enrolled in the IPACOVID trial [73]. A total of 36 patients participated with safety and feasibility being the aims of this multi-centered nonrandomized prospective study. Primary endpoint was an increase in arterial oxygen partial pressure (PaO_2_) to the fractional inspired oxygen (FiO_2_) (PaO_2_/FiO_2_) ratio or the pulse oximetry saturation (SpO2) to FiO2 (SpO2/FiO2) ratio of at least 20% at 24h following LDRT in at least 30% of evaluable patients. Secondary endpoints were mortality, radiological improvement, and inflammatory markers. Patients included had moderate/severe pneumonia and were not candidates for ICU admission due to medical comorbidities or poor performance status. A single dose of 50 cGy was given with a 3D conformal technique with two anterior–posterior and posterior–anterior (AP PA) fields. A total of 50% of evaluable patients showed significant improvement of SpO_2_/FiO_2_ ratio with a mean percentage of 38.82%. Improvement in C-reactive protein (CRP) was also observed. Patients who died from COVID-19 pneumonia following LDRT were those with poor prognostic factors on admission. Therefore, authors suggest that LDRT should be delivered earlier rather than later in advanced stages.

#### 2.4.3. Cancer Risks Due to Whole Lungs LDRT with Different Techniques

For the risk estimation of RICand cardiac disease, RT plans for whole lung irradiation were designed either on patient CT images or on reference phantoms [8,74,75]. Table A4 summarizes the LDRT techniques used and the parameters taken into account for the estimation of RIC from published studies. Data include estimations for the lifetime attributable risk (LAR) of developing RIC, especially lung cancer, LAR of cancer mortality, and LAR of cardiovascular disease. Risk estimates were generated considering relevant risk factors, namely cancer site, sex, age at exposure, cigarette smoking, and baseline heart disease risk. The main dosimetric objective for the RT plans was the irradiation of at least 90–95% of planning target volume (PTV) with 95% of the prescribed dose. PTV included both lungs plus a margin of 0.5 cm in all directions [76].

Studies presented low cancer risks for all the radiotherapy techniques, with IMRT and VMAT techniques inducing lower overall risks. Table A5 presents the main results of the studies analyzed in Table A4. The mortality risk assessments are similar to the cancer risk results.

Lung is the organ receiving the highest dose from whole-lung irradiation for both sexes, and therefore the highest LAR values. The organ presenting the second-highest LAR for females is the breast, especially for exposures of young women, while for males it is the heart. Organs outside the treatment field result in an extremely small risk of cancer induction. These risk estimates become higher when taking into account baseline risk factors, such as high serum cholesterol level, elevated systolic blood pressure, smoking, and family history of myocardial infarction before 60 years old [74]. Shuryak points out that the combined LAR of radiation-induced lung cancer and heart disease associated with LDRT for COVID-19 can exceed 5%/Gy in some patient cohorts, such as young (<60 years old) smokers with high baseline heart disease risk factors.

All studies agree to the RIC incidence and mortality risks for the female population. While differences of dose in organs between males and females are small, mainly due to structure and volume differences, the total risks are higher for females. Hernandez reports the LAR for females to be on average 2.2 times higher [76]. That incidence is due to the higher probability of cancer incidence for females in cancer estimation models. Moreover, breast, as the women’s high radiosensitive organ, lies fully or partially within the large treatment field in different delivery techniques and contributes to the increase in the total risk. Another moderating factor is the age at the time of exposure. At lower ages, LAR has higher values and also higher differences among different techniques, namely 3D-CRT or IMRT/VMAT techniques. The cancer risk differences among different delivery techniques are reduced when increasing the age of exposure [75].

Comparing the different delivery techniques, studies showed that LAR for lung cancer is statistically similar for all the delivery techniques, while LAR for cancer of organs located close to the lung like breast, and stomach are significantly higher in 3D-CRT techniques [8,75]. The superiority of intensity-modulated techniques may be related to the radiation modulation, which could better spare the organs at risk. Moreover, the calculated conformity indexes (CI) were found to be similar for all the techniques, while the homogeneity indexes (HI) were better in IMRT and VMAT plans [75].

In all the delivery techniques, LDRT cancer risk analysis showed that RT plans with higher prescribed doses resulted in higher cancer risks, because the Biological Effects of Ionizing Radiation-VII (BEIR-VII) report model, used by LDRT studies, considers a linear relationship between the organ doses and cancer risks in low doses up to 200 cGy [8,74,75]. Studies performed the risk estimations for prescribed doses of 50–150 cGy, with Arruda observing an unacceptable or cautionary LAR for lung cancer in all women and men <60 years with an RT dose >100 cGy [8].

## 3. Discussion

Presently, the ongoing COVID-19 epidemic requires the utmost attention of health institutions and responsible organizations on a worldwide scale. Amidst this perilous situation, effective and rapid triage of the incoming patients’ flow will decrease the pressure induced on healthcare facilities in their fight against the disease. RT-PCR tests might be the gold standard for detecting the virus; however, they have proven to be time-consuming in terms of obtaining the results and, in many cases, they present shortages, especially within countries with few resources [33,77,78]. Another disadvantage of virus tests is that their already low sensitivity may be compromised by improper specimen sampling, handling, and transferring [21,79]. CT can play an important role as a reliable supplementary method to RT-PCR for diagnosis of COVID-19, especially in cases where tests might provide many false-negative results, or in countries that cannot afford sufficient kit supplies to their medical centers [22,43,77,78,80]. However, COVID-19 has a high transmission rate and can easily spread via patient and imaging equipment [33,42]. Therefore, the creation of a method to reduce virus transmission inside healthcare facilities would be significant. Much future research based on the creation of interim protocols and safety measures may move in that direction. Another crucial factor of CT examination is the radiological risk it may induce on the population [21,22]. A research conducted in 2020 by Cristofaro et al. concluded that not only do COVID-19-infected patients receive higher dose levels than uninfected ones, but also young patients recorded the highest dose levels from CT examinations among other age groups [81]. These significant findings can move research towards young patient monitoring and accurate assessment of radiation impact.

Comparing chest CT and RT-PCR assays for COVID-19 detection, Karam et al. [82] carried out a comprehensive systematic review [82]. This review evaluated and presented data from 13 previously conducted comparative studies. The authors concluded that 79.2% of patients who initially tested negative for COVID-19 (but later tested positive) had a CT scan indicative of COVID-19. They concluded that chest CT scans are capable of detecting the majority of RT-PCR diagnosed cases that initially tested negative and subsequently positive for COVID-19 [82]. Additionally, when compared to RT-PCR tests, low-dose chest CT demonstrated superior sensitivity, specificity, positive predictive value, negative predictive value, and accuracy for COVID-19 diagnosis in emergency room patients, and especially in patients with clinical symptoms lasting more than 48 h. Disease likelihood increased from 43.2% (pre-test probability) to 91.1% or 91.4% (post-test probability) in patients with a positive CT result, while in patients with a negative CT result, the likelihood of disease was reduced to 9.6% or 3.7% for all patients or for those patients experiencing clinical symptoms for longer than 48 h [83]. In another study conducted by Bahrami-Motlagh et al. [43], a low-dose chest CT scan demonstrated high sensitivity and a negative predictive value for the identification of COVID-19 when compared to the initial RT-PCR test as the gold standard. Sensitivity, specificity, positive predictive values, negative predictive values, and accuracy of low-dose chest CT scans were 96.6%, 36.5%, 64.7%, 90%, and 69.3%, respectively, based on positive RT-PCR results [43].

Similar to other viruses, the SARS-CoV-2 virus continually mutates, thus constantly creating new variants. Numerous studies have suggested that this genetic progression and mutations resulting in several COVID-19 variants may impair the sensitivity of RT- PCR diagnostic kits, with even a few mutations resulting in a significant decrease in sensitivity and an increased number of false-negative RT-PCR results [84]. On the other hand, there is heterogeneity in the literature regarding chest-CT imaging findings amongst different variants. One study found that there was no significant difference in chest CT imaging findings between the variant and non-variant groups, suggesting that clinical and laboratory characteristics should take precedence over chest CT results when it comes to differentiating patients infected with SARS-CoV-2 variations from those infected with the non-variant strain [85]. A study by Cheng et al. found that the chest CT imaging findings in children infected by the Delta variant were milder in comparison with those of the original strain. Finally, another study documented both differences and similarities in the chest CT findings of the patients from the first wave and the second wave of infection and concluded that there is unpredictability among the various COVID-19 strains concerning the severity of the radiological abnormalities obtained [86,87]. As a result, future studies addressing this matter and comparing RT-PCR sensitivity and specificity to that of chest-CT imaging findings for the diagnosis of specific COVID-19 variants would be of great interest.

Homayounieh et al., in their international study of CT utilization on COVID-19 patients, concluded that there is an urgent need for the establishment of CT frequency guidelines and specific scan protocols, which will minimize side-effects of a cumulative effective dose from multiple CT exams [42]. However, this research had a limited sample size of patients per site. Future research with more patients, as well as more participating sites, should investigate CT implementation worldwide thoroughly. Those studies should conclude with a way of applying safety protocols in daily routine workflow globally, taking into account clinical features and disease severity of COVID-19 patients.

Preliminary evidence suggests that LDRT offers some benefit as a treatment modality against COVID-19 pneumonia. Single fraction LDRT appears feasible with mild acute toxicity. On the other hand, all trials included a small number of patients, short follow-up. and heterogeneous patient cohort in terms of comorbidities, functional status. and previous anti-COVID-19 treatment. All these confounding factors make it crucial to obtain more robust evidence, through randomized phase III trials, to confirm its benefit.

In terms of fractionation schemes, doses from 50 to 150 cGy, as a single or two fractions in the aforementioned trials, appear to be safe and effective, however more evidence is needed to specify the exact scheme, as more than 100 cGy may exacerbate inflammatory response according to Ameri. The optimal technique has yet to be clarified, as critically ill patients need to stay outside ICU for as short a period of time as reasonably possible and the 2D technique offers this advantage. The timing of LDRT delivery is also crucial. One possible explanation for Papachristofilou’s negative results could be the late delivery of radiation, as patients have already very advanced disease. Early consideration of LDRT was also suggested by Arena. Finally, the use of LDRT along with other anti-COVID-19 treatments remains to be studied and may lead to conclusions about complementary actions, possible interactions, and appropriate sequencing of treatment. More light will be shed by ongoing trials (Table A3) and results are very much anticipated.

At the time of writing this review, a trial (Hess et al.) has transitioned to its phase 3 component, and should it confirm the positive results of its phase 2 component, it could lead to a significant change in the current management of COVID-19 patients [71]. Furthermore, should a positive impact be proven from new trials, LDRT might regain the interest lost for more than half a century, as an option for other viral or atypical types of pneumonia, especially when no other therapeutic options exist. Apart from COVID-19, this novel idea of LDRT has been investigated for various inflammatory and degenerative conditions, such as osteoarthritis and enthesopathy, and shows promising results as evidenced by recent prospective cohort studies [88,89,90,91]. Finally, lessons learned from these trials could be applied to future pandemics as LDRT does not discriminate against viruses.

Historical review for LDRT does not provide evidence for morbidity or mortality associated with post-infection radiation exposure, thus the possible potential risks from low-dose lung irradiation remain unclear [92,93,94]. Therefore, the quantitative comparison of benefits and risks will assist clinical institutions in the decision of including it in the treatment management system.

As the need to reduce risk is of particular importance for younger patients, especially females and patients with high baseline risk factors, modulated therapies seem to be better choices. Nevertheless, a simple AP-PA treatment method provided relatively similar conformity indexes with the modulated techniques and may be appropriate for initial clinical trials. It should also be considered that IMRT/VMAT are expensive, more time-consuming techniques, and make the patients’ time in the machine longer. For either technique, the risk for development of radiation pneumonitis is not examined, as the LDRT dose is much lower than that required for radiation-induced pneumonitis. A review from Zhang refers a mean lung dose of 14 Gy and V_5 Gy_ > 40% (lung volume exceeding 5 Gy, over 40%) for radiation pneumonitis incidence [95].

In order to quantify the effect of delivering the dose in one or two fractions on the LAR for lung cancer, Schneider’s model, a more sophisticated dose–response model, was used [96,97]. Notably, the LAR found for a single fraction was lower than for two fractions for both sexes and all ages [76]. That indicates that, for the low dose range of LDRT, fractionation has a minor effect on carcinogenesis. Additionally, LDRT for COVID-19 with a single dose is both cost- and time-effective.

Comparing the LDRT effective dose with other radiological techniques, it appears to be of the same order of magnitude with the highest dose interventional radiological techniques, such as transjugular intrahepatic portosystemic shunt placement (180 mSv) and abdominal aortic endoprosthesis (166 mSv) [76]. Sodickson estimated the cumulative effective doses and LAR values for adult patients from CT scans performed throughout their life at a tertiary care academic medical center [98]. A total of 15% received comparable estimated cumulative effective doses or higher than COVID-19 LDRT effective doses, while associated mean and maximum LAR values of 0.3% and 12% for cancer incidence were within the COVID-19 LDRT LAR range.

## 4. Conclusions

The COVID-19 pandemic that humanity has been dealing with for the last two years has brought the scientific community, and more particularly healthcare professionals, unprecedented challenges. RT-PCR tests might be the gold standard for detecting the disease, but there is an urgent need for better triage of patients. CT can play an important diagnostic role, but not without a plan. Estimates of radiation-induced lifetime cancer risk, mainly for lung and breast, and risk for heart disease, are also needed to quantify the benefit–risk balance of radiation treatment for COVID-19. In this rapidly changing environment, answers from ongoing studies are pressingly needed, as the COVID-19 pandemic might complete its cycle before results become available, emphasizing the need for rigorous and effective scientific research.

## Data Availability

Not applicable.

## Appendix A

**Table A1:** Literature studies monitoring LDCT/uldCT protocol implementation.

Study	No of pts	Protocol	Effective Dose (mSv)
Tabatabaei et al., 2020 [21]	20	HDCT/LDCT	6.60/1.80
Mohan et al., 2020 [99]	141	HDCT/LDCT	6.33/1.45
Bahrami et al., 2021 [43]	163	HDCT/LDCT	5.8/0.91
Desmet et al., 2021 [77]	610	LDCT	0.74
Leger et al., 2020 [39]	80	LDCT	0.60
Samir et al., 2021 [100]	250	uldCT	0.59
Zarei et al., 2020 [101]	36	uldCT	0.50

HDCT: High Dose CT, LDCT: Low Dose CT, No: Number, pts: patients, uldCT: Ultra Low Dose CT.

**Table A2:** LDRT published trials.

Name	Author	Country	Type of Study	Dose (cGy/fx)	Clinical Benefit as per Primary Endpoint
LOWRAD	Sanmamed [17]	Spain	I-II single-arm	100/1	yes
-	Ameri [18]	Iran	I-II, single-arm	50/1 ± 50/2 or 100/1	yes
RESCUE 1–19 (elderly)	Hess [71]	USA	I-II, transitioned to III	150/1	yes
COVID-RT-01 (critically ill)	Papachristofilou [72]	Switzerland	II, double-arm	100/1	no
IPACOVID (not ITU candidates)	Arenas [73]	Spain	I, transitioned to II	50/1 ± 50/2	yes

fx: fraction, ICU: intensive care unit, LDRT: low dose radiation therapy.

**Table A3:** LDRT ongoing trials.

Trials	Dose Scheme (cGy)	Est Completion/Update
COLOR 19 Brescia Italy N: 30, Monocentric single arm [102]	70	August 2022
PREVENT Ohio, USA N: 100, phase II NCT04466683 [103]	35 100	2022
COVRTE-19 Spain (bad prognosis pts) N: 41, single-arm NCT04414293 [104]	unknown	unknown
LOCORAD India N: 20, case control study NCT04904783 [105]	50	December 2021
RESCUE 1-19 USA N: 52, phase III NCT04433949 [106]	150	2022
ULTRA-COVID Spain N: 15, single arm NCT04394182 [107]	80	2021
Lancashire, UK, phase I N: 13, Feasibility NCT04572412 [108]	50 ± 50	2021
VENTED COVID Ohio, USA N: 24, phase II NCT04427566 [109]	80	December 2021
Madrid, Spain N: 96, phase II, LDRT vs. pharmacological Rx NCT04380818 [110]	50 ± 50	2021
Anti-inflammatory effect Mexico N: 30, randomized double-blinded NCT04534790 [111]	100	completed/not published

Est: Estimated, LDRT: low dose radiation therapy, pts: patients, Rx: prescription.

**Table A4:** LDRT techniques used and methodology adopted for the assessment of radiation-induced risk factors.

Study	No of pts	Age	RT Technique	Calculated	Estimated	Organs Taken into Account	Prescribed Dose (cGy)	Risk Factors
Banaei [75]	32 COVID pts	32–74 y	3D-CRT AP-PA	organ mean/max doses	RIC risks	lungs	100	cancer site
						heart		
			3D-CRT 8 fields	CI		breast		gender
						liver		
			IMRT- 8 fields		radiation induced mortality risks	stomach		age at exposure
				HI		thyroid		
			VMAT- 2 full arcs			esophagus		time elapsed after exposure
						spinal cord		
Arruda [8]	simulation from a median female body	20–80 y	3D-CRT AP-PA	organ mean doses	lifetime RIC risks	lung	50	cancer site
						breast	70	
			IMRT- 7 fields		cardiovascular REID due to ischemic heart disease	liver	100	gender
						esophagus		
						heart	150	age at exposure
Hernandez [76]	reference male and female ICRP phantoms	Adults	3D-CRT AP-PA	effective dose as the tissue-weighted sum of the equivalent dose in all specified tissues and organs	RIC incidence by cancer site	lungs	50	cancer site
						heart		
						breast		
						liver		
						stomach	70	gender
						thyroid		
						esophagus		
						spinal cord		
					total RIC obtained by effective dose	brain		
						salivary glands		
						colon	100 in 2 fx	age at exposure
						gonads		
						bladder		
						skin		
						prostate		
						uterus		
Shuryak [74]	24	50–85 y	whole-lung irradiation	-	lifetime RIC risk	lung	50	gender
								age at exposure
					heart disease risks	heart	100	cigarette smoking
							150	baseline heart disease

3D-CRT: 3-dimentional-conformal radiotherapy, AP-PA: Anterior Posterior–Posterior Anterior, CI: conformity index, fx: fractions, HI: Homogeneity index, ICRP: International Commission on Radiological Protection, IMRT: Intensity modulated radiation therapy, LDRT: low dose radiation therapy, No: number, pts: patients, REID: risk of exposure-induced death, RIC: radiation induced cancer, RT: Radiotherapy, VMAT: Volumetric modulated arc therapy.

**Table A5:** Results for the radiation-induced risk assessment. The main endpoints from each study are presented.

Study	Organs with Highest Radiation Doses	Organ Dose Differences between Males and Females	Highest RIC Risk	RIC Risk for Other Organs	LAR vs. Age	LAR vs. Sex	Other
Banaei [75]	lung	small (except the breast)	lung (for all delivery techniques statistically similar)	breast, and stomach significantly higher in 3D-CRT techniques compared to IMRT or VMAT	at lower ages:	female > male	CI: similar in all techniques
	heart				higher LAR values		
	breast (for females)				higher differences among different techniques		HI: IMRT ≈ VMAT (better) <3D-CRT
Arruda [8]	lung	-	lung	second highest for females: breast	at lower ages: higher LAR values	female > male (except for liver)	Dose > 100 cGy unacceptable or cautionary
	heart						
	breast (for females)						
Hernandez [76]	lung	small (except the breast)	lung	second highest for females: breast	at lower ages: higher LAR values	female > male	fractionation scheme: negligible effect on RIC risk
	heart			second highest for males: heart			
	breast (for females)						
Shuryak [74]	lung	-	lung	second highest: heart	at lower ages: higher LAR values	female > male	lung RIC and heart disease risks higher in: smoking patients & patients with cardiac risk factors
	heart						

3D-CRT: 3-dimensional-conformal radiotherapy, CI: Conformity index, HI: Homogeneity index, IMRT: Intensity modulated radiation therapy, LAR: lifetime attributable risk, RIC: radiation-induced cancer, VMAT: Volumetric modulated arc therapy.
